# Patterns of pharmacological treatment in patients with atrial fibrillation: an analysis from the prospective GLORIA-AF Registry Phase III

**DOI:** 10.1186/s12916-025-03858-w

**Published:** 2025-01-21

**Authors:** Bernadette Corica, Giulio Francesco Romiti, Giuseppe Boriani, Brian Olshansky, Tze-Fan Chao, Menno V. Huisman, Marco Proietti, Gregory Y. H. Lip, on behalf of the GLORIA-AF Investigators

**Affiliations:** 1 Liverpool Centre for Cardiovascular Science at University of Liverpool, Liverpool John Moores University and Liverpool Heart & Chest Hospital, Liverpool, UK; 2 Cardiology Division, Department of Biomedical, Metabolic and Neural Sciences, University of Modena and Reggio Emilia, Policlinico Di Modena, Modena, Italy; 3 Department of Translational and Precision Medicine, Sapienza – University of Rome, Rome, Italy; 4 Division of Cardiology, Department of Medicine, University of Iowa Hospitals and Clinics, Iowa City, Iowa, USA; 5 Division of Cardiology, Department of Medicine, Taipei Veterans General Hospital, Taipei, Taiwan; 6 Institute of Clinical Medicine, and Cardiovascular Research Center, National Yang Ming Chiao Tung University, Taipei, Taiwan; 7 Department of Thrombosis and Hemostasis, Leiden University Medical Center, Leiden, the Netherlands; 8 Department of Clinical Sciences and Community Health, University of Milan, Milan, Italy; 9 Division of Subacute Care, IRCCS Istituti Clinici Scientifici Maugeri, Milan, Italy; 10 Department of Clinical Medicine, Aalborg University, Aalborg, Denmark

**Keywords:** Atrial Fibrillation, Comorbidities, Multimorbidity, Clinical Complexity, Latent Class Analysis

## Abstract

**Background:**

Polypharmacy (i.e., treatment with ≥ 5 drugs) is common in patients with atrial fibrillation (AF) and has been associated with suboptimal management and worse outcomes. Little is known about how prescribed drug patterns affect management and prognosis in patients with AF.

**Methods:**

Based on data from the prospective global GLORIA-AF Registry Phase III (recruiting patients with AF and CHA_2_DS_2_-VASc score ≥ 1), we performed a latent class analysis to identify treatment patterns based on 14 drug classes including cardiovascular (CV) and non-CV drugs. We analysed associations with oral anticoagulant (OAC) use and risk of a composite primary outcome (all-cause death and major adverse cardiovascular events (MACE)) and secondary outcomes.

**Results:**

Among 21,245 patients (mean age 70.2 ± 10.3 years, 44.9% females), we identified 6 patterns: i) Low Medicated pattern (18.3%); ii) Hypertension pattern (21.1%); iii) Heart Failure pattern (20.0%); iv) CV Prevention pattern (21.0%); v) Mixed Morbidity pattern (4.5%); and vi) High Medicated pattern (15.0%). All groups had higher odds of OAC use vs the Low Medicated pattern, with highest prevalences in the Heart Failure pattern (OR [95%CI]: 2.17 [1.90–2.48]) and the High Medicated pattern (OR [95%CI]: 2.08 [1.77–2.44]). Over 3-year follow-up, Heart Failure, Mixed Morbidity and High Medicated patterns were associated with higher risk of the primary composite outcome (aHR [95%CI]: 1.32 [1.14–1.53]; 1.45 [1.17–1.80] and 1.35 [1.14–1.60], respectively). Similar results were observed for all-cause mortality.

**Conclusions:**

In patients with AF, different treatment patterns can be identified. Each pattern was associated with unique OAC use and long-term clinical outcomes.

**Supplementary Information:**

The online version contains supplementary material available at 10.1186/s12916-025-03858-w.

## Background

Patients with atrial fibrillation (AF) often have concurrent diseases. Recent epidemiological studies showing an increasing burden of comorbidities at time of AF diagnosis [1], with a “polypharmacy” state that can follow. While the definition of polypharmacy and its estimates vary [2, 3], up to 40% of patients with AF are treated with 5 or more drugs [4]. Polypharmacy has been linked to detrimental effects on the prognosis of patients with AF, including all-cause mortality, thromboembolism and major bleeding [4, 5]. Indeed, concomitant pharmacological treatment contributes to increased complexity of management of patients with AF [6].

Indeed, in older adults, medication patterns have been previously described in association with various diseases [7]. In patients with AF, previous studies have shown how clinical phenotypes can be characterised according to comorbidities [8, 9], but whether phenotypic patterns of treatments can be identified in these patients with AF is unclear.

In this exploratory analysis from *Global Registry on Long-Term Antithrombotic Treatment in Patients with Atrial Fibrillation* (GLORIA-AF) Phase III Registry, we aimed to i) identify phenotypic patterns of treatment in patients with AF; and ii) analyse associations between phenotypic patterns and prognosis of patients with AF.

## Methods

We used data from the phase III of the GLORIA-AF study, a prospective, international registry programme structured in 3 phases, with the primary aim of assessing the long-term real-world safety and efficacy of dabigatran etexilate in patients with AF. Full details on the design of GLORIA-AF registry have been described elsewhere, along with primary results [10–13].

In short, during phase III (2014–2016), patients (≥ 18 years), with recently diagnosed non-valvular AF (i.e. within 3 months, or 4.5 months in Latin America), and CHA_2_DS_2_-VASc score ≥ 1 were consecutively enrolled, with a planned 3-year follow-up. Main exclusion criteria were AF due to a reversible cause, presence of mechanical heart valve (or patients expected to undergo valve replacement), having received VKA for more than 60 days during lifetime, or presence of other clinical indications for treatment with OAC, and limited life expectancy (< 1 year).

The study, conducted according to the Good Clinical Practice and the Declaration of Helsinki, was approved by local institutional review boards at each participating centre. All patients provided written informed consent. Data on demographics, comorbidities and treatment for each patient were collected at baseline and recorded by investigators using standardised case report forms (CRF). For this analysis, we included patients with complete data on the treatments included in the analysis and with complete information on follow-up for the primary composite outcome (see below).

### Treatments, comorbidities and clinical variables

We focused on 14 classes of pharmacological treatments recorded, among others, in the CRF at baseline. Drug classes were selected according to data availability, clinical relevance, and overall prevalence of use in our cohort. We included cardiovascular drugs (angiotensin converting enzyme inhibitors (ACEi)/angiotensin receptor blockers (ARB); beta-blockers; diuretics; digoxin; verapamil/diltiazem; class IC antiarrhythmics [i.e. propafenone and flecainide]; amiodarone/dronedarone, and other antihypertensive agents [including alpha blockers and vasodilators]), and non-cardiovascular drugs (statins and non-statin lipid lowering drugs, oral hypoglycemic drugs, insulin, selective serotonin reuptake inhibitor (SSRI)/other antidepressants, and proton pump inhibitor/H2 receptor blockers). All the drug categories were defined as reported by the investigators in the CRF.

Polypharmacy was considered concurrent use of ≥ 5 drugs among the categories reported above; multimorbidity was ≥ 2 comorbidities. We additionally considered the use of antithrombotic drugs (vitamin K antagonist (VKA) or non-vitamin K antagonist oral anticoagulants (NOAC), antiplatelets, or none). Data on demographics and major comorbidities were included in the analysis as collected by the investigators in the CRF. Classes were named according to their most relevant clinical characteristics and to the presence of polypharmacy.

### Follow-up and outcomes

During phase III, patients enrolled underwent a 3-year follow-up, in which data regarding the occurrence of major clinical outcomes were collected. For this analysis, we considered the following outcomes: i) all-cause mortality; ii) major adverse cardiovascular events (MACE; which included cardiovascular death [death ascribed to cardiovascular causes as recorded by investigators], stroke, and myocardial infarction); iii) thromboembolism (the composite of stroke, transient ischemic attack, and other non-central nervous system thromboembolism); iv) major bleeding (defined as a life-threatening or fatal bleeding, symptomatic bleeding in a critical organ, or a bleeding associated with a haemoglobin reduction of ≥ 20 g/L or leading to ≥ 2-unit of blood transfusion). For this analysis, we defined our primary outcome as the composite of all-cause death and MACE; we analysed the other events individually as exploratory secondary outcomes.

### Statistical analysis

To identify patterns of treatment in our cohort, we performed an exploratory latent class analysis (LCA) based on the 14 treatments defined above, using the ‘poLCA’ package in R [14]; use of antithrombotic treatment was not included among the variables used for the LCA, and was subsequently analysed according to the treatment patterns identified. The optimal number of classes was selected considering the Bayesian Information Criterion (BIC) and the consistent Akaike Information Criterion (cAIC), with lower values indicating better fit [15], and also according to clinical judgment. For subsequent analyses, we then assigned each patient included to one of the latent classes, according to the modal posterior probability of membership. The groups were then named and described after evaluation of the most relevant clinical characteristics; descriptive statistics were then computed and reported according to the latent classes’ allocation.

Mean and standard deviation (SD), or median and interquartile range [IQR] were used to report continuous variables; parametric tests were used to compare normally distributed variables, while non-normally distributed variables were compared using non-parametric tests. Binary and categorical variables were reported according to frequencies and percentages, and compared with chi-square test.

We analysed the association between the latent classes and use of antithrombotic treatment using a multiple logistic regression model; components of CHA_2_DS_2_-VASc score (age < 65, 65–75 or ≥ 75 years, sex, hypertension, diabetes, heart failure, coronary artery disease, history of stroke/transient ischemic attack and peripheral artery disease), body mass index, type of AF (paroxysmal, persistent or permanent), and history of previous bleeding were included as covariates. We reported results as Odds Ratios (OR) and 95% Confidence Intervals (CI).

The association of the latent classes with risk of major outcomes was assessed using multiple Cox-regression models, with the same covariates used in the logistic regression model, and additionally adjusted for the use of OAC. Results were reported as Hazard Ratios (HR) with 95% CI. For the primary composite outcome, we also performed and reported Kaplan–Meier curves, and compared survival distributions with the log-rank test. A two-sided *p* < 0.05 was considered statistically significant. All the analyses were performed using R 4.3.1 (R Core Team 2020, Vienna, Austria).

## Results

Overall, 21,245 patients enrolled in the GLORIA-AF Registry phase III were included in this analysis (mean age 70.2 ± 10.3 years, 44.9% females). Metrics (BIC and cAIC) for the LCA model using 2–6 classes are reported in Additional File 1: Table S1; based on those metrics and clinical judgment, the model with 6 classes was selected for further analyses.

### Patterns of treatments according to latent classes

**Fig. 1:**
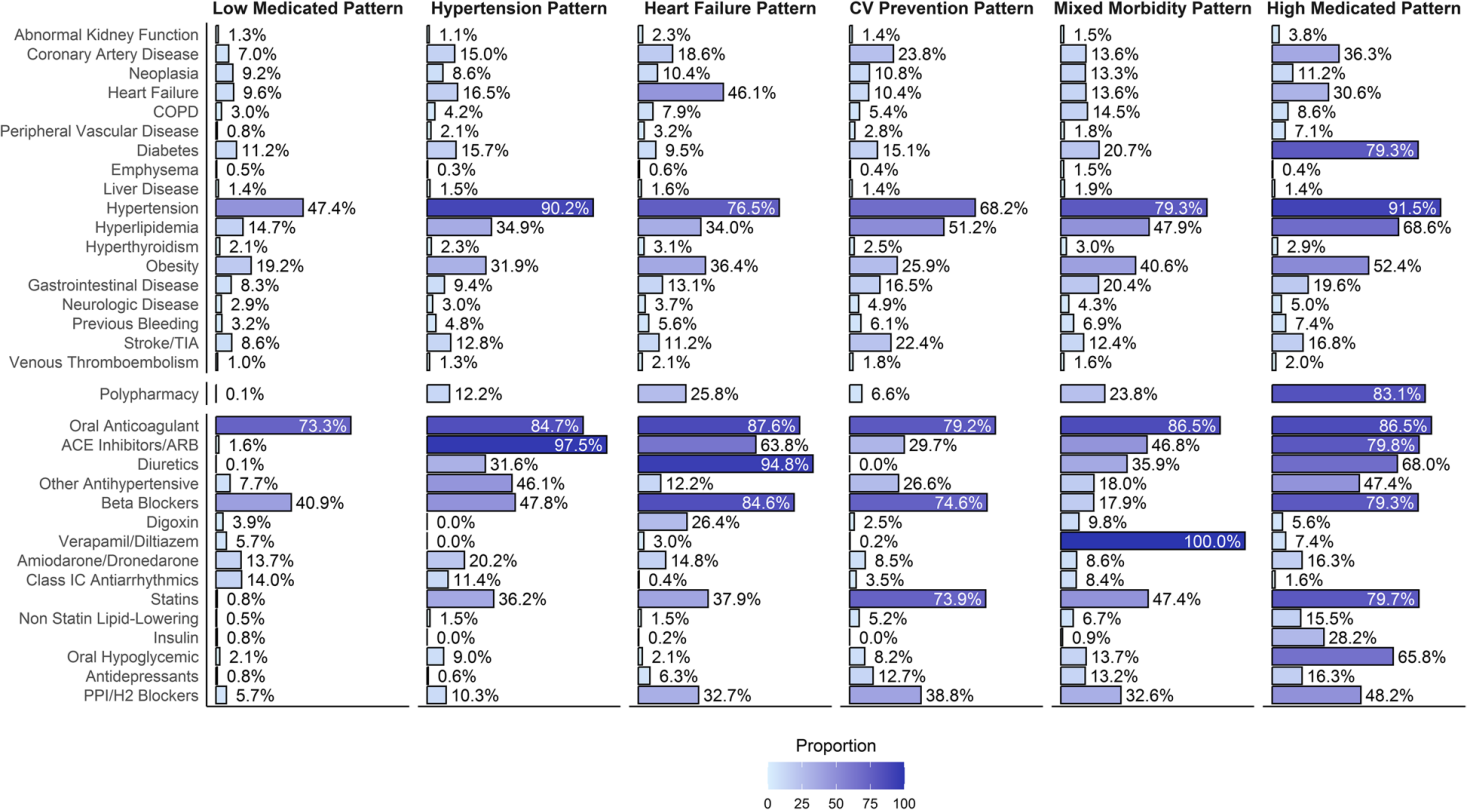
Prevalence of drug classes and comorbidities according to the latent classes. Legend: ACE = Angiotensin Converting Enzyme; ARB = Angiotensin Receptor Blocker; COPD = Chronic Obstructive Pulmonary Disease; TIA = Transient Ischemic Attack

Baseline characteristics of patients included in each of the latent classes are reported in Additional File 1: Table S2; a graphical representation showing the prevalence of comorbidities and treatment use for each drug is reported in Fig. 1.

We identified a group of Low Medicated patients (*n* = 3,888, 18.3%), which was characterized by an overall low use of all drugs classes, except for beta-blockers (40.9%) and a consistent low prevalence of most comorbidities, with 47.4% having arterial hypertension.

The numerically largest group was represented by the Hypertension pattern (*n* = 4,491, 21.1%), in which 90.2% of patients had arterial hypertension, and with high prevalence of use of ACEi/ARB (97.5%) as well as other cardiovascular drugs.

In the Heart Failure pattern group (*n* = 4,259, 20.0%), treatment phenotype was characterised by a high prevalence of drugs used for heart failure, such as diuretics (94.8%), beta-blockers (84.6%) and ACEi/ARB (63.8%), consistent with the highest prevalence of heart failure (46.1%) observed among the groups. Conversely, the CV Prevention pattern (*n* = 4,465, 21.0%) was mostly characterised by a high proportion of statins and beta-blockers use (73.9% and 74.6%, respectively), with a significant proportion of patients showing CAD, previous stroke/TIA, and hyperlipidemia.

Finally, we observed a Mixed Morbidity phenotype (*n* = 955, 4.5%), with relevant proportion of patients with both cardiovascular and non-cardiovascular comorbidities and characterized by the highest use of verapamil/diltiazem, as well as other cardiovascular and metabolic drugs, and a High Medicated pattern (*n* = 3,187, 15.0%) which showed highest prevalence of use of several drugs, including antidiabetics, lipid-lowering drugs, proton pump inhibitors (PPI)/Histamine H2 (H2)-receptor blockers and other cardiovascular drugs; this group of patients had the highest prevalence of diabetes (79.3%), hypertension (91.5%), hyperlipidemia (68.6%) and obesity (52.4%), and 83.1% were polymedicated (≥ 5 drugs). Similarly, multimorbidity was highest in this group (98.7%).

### Use of antithrombotics according to treatment patterns

Treatment with OAC was largely prevalent in all groups, with highest figures observed in the Heart Failure pattern (87.6%) and lower prevalence observed in the CV Prevention and Low Medicated patterns (79.2% and 73.3%, respectively) (Additional File 1: Figure S1). NOACs were more frequently found in the Mixed Morbidity pattern (67.2%), while 14.5% of patients in the Low Medicated pattern did not receive any antithrombotic treatment.

On multiple logistic regression analysis, compared to the Low Medicated Pattern, all groups were associated with higher odds of receiving OAC (*p* < 0.001 for all groups; Table 1). Conversely, no statistically significant association was found for the use of NOACs vs. VKA.

**Table 1:** Logistic Regression for Treatment Prescription according to latent classes

**Group**	**OAC Use (vs. No OAC)** OR [95%CI]	**NOAC Use (vs. VKA)** OR [95%CI]
Low Medicated Pattern	Ref	Ref
Hypertension Pattern	**1.77 [1.57–2.00]**	0.89 [0.79–1.01]
Heart Failure Pattern	**2.17 [1.90–2.48]**	0.89 [0.79–1.01]
CV Prevention Pattern	**1.38 [1.23–1.54]**	0.98 [0.87–1.11]
Mixed Morbidity Pattern	**2.01 [1.63–2.47]**	1.17 [0.96–1.42]
High Medicated Pattern	**2.08 [1.77–2.44]**	0.93 [0.80–1.08]

*CI* Confidence Interval, *CV* Cardiovascular, *OAC* Oral Anticoagulant, *NOAC* Non-vitamin K antagonist oral anticoagulant, *VKA* Vitamin K antagonist. Bold values depict results with *p* < 0.05

### Risk of adverse outcomes

**Fig. 2:**
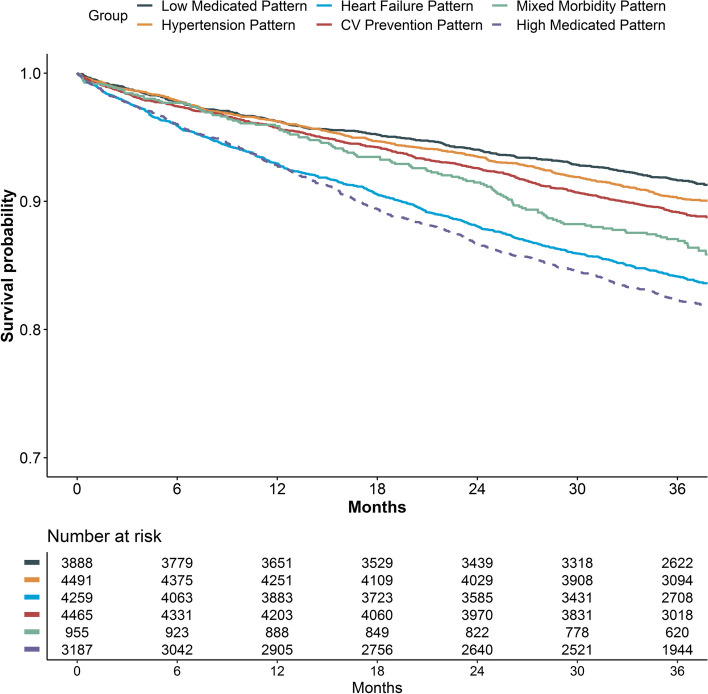
Kaplan–Meier curves for the primary composite outcome of all-cause death and MACE according to latent classes. Legend**:** Log-Rank *p* < 0.001

Kaplan–Meier analysis for the primary composite outcomes according to treatment patterns are reported in Fig. 2. After a median follow-up of 3.0 [IQR: 2.9–3.1] years, incidence of the composite outcome of all-cause death and MACE was highest in the High Medicated and Heart Failure pattern; the Low Medicated pattern showed instead the highest survival probability (Log-rank *p* < 0.001 for all groups compared to the Low Medicated pattern except Hypertension pattern, *p* = 0.053).

Results of the multivariate Cox-regression models for all outcomes analysed are reported in Table 2. Compared to the Low Medicated pattern, we observed a higher hazard of the primary composite outcome in the Heart Failure pattern (HR [95%CI]: 1.32 [1.14–1.53]), the Mixed Morbidity pattern (HR [95%CI]: 1.45 [1.17–1.80]) and the High Medicated pattern (HR [95%CI]: 1.35 [1.14–1.60]), while no statistically significant results were observed for the other groups.

**Table 2:** Multiple Cox Regressions on the Risk of Major Outcomes according to latent class allocation

	**Low Medicated Pattern**	**Hypertension Pattern**	**Heart Failure Pattern**	**CV Prevention Pattern**	**Mixed Morbidity Pattern**	**High Medicated Pattern**
**Primary Outcome**						
Composite of All Cause Death and MACE						
*IR [95%CI]*	3.0 [2.6–3.3]	3.4 [3.1–3.7]	5.8 [5.4–6.3]	3.8 [3.5–4.2]	4.7 [3.9–5.6]	6.6 [6.1–7.2]
*aHR [95%CI]*	Ref	0.95 [0.81–1.11], *p* = 0.489	**1.32 [1.14–1.53], *p* < 0.001**	1.04 [0.89–1.20], *p* = 0.648	**1.45 [1.17–1.80], *p* = 0.001**	**1.35 [1.14–1.60], *p* = 0.001**
**Secondary Outcomes**						
All Cause Death						
*IR [95%CI]*	2.2 [1.9–2.4]	2.5 [2.2–2.8]	4.8 [4.4–5.2]	2.7 [2.4–3.0]	3.8 [3.1–4.6]	5.0 [4.5–5.5]
*aHR [95%CI]*	Ref	0.94 [0.78–1.12], *p* = 0.472	**1.41 [1.19–1.68],** ***p* < 0.001**	1.00 [0.84–1.19], ***p* = 0.982**	**1.61 [1.26–2.05],** ***p* < 0.001**	**1.35 [1.11–1.64],** ***p* = 0.003**
MACE						
*IR [95%CI]*	1.6 [1.4–1.8]	1.8 [1.5–2.0]	3.0 [2.7–3.3]	2.2 [1.9–2.4]	2.1 [1.6–2.8]	3.6 [3.2–4.0]
*aHR [95%CI]*	Ref	0.87 [0.70–1.08], *p* = 0.209	**1.24 [1.01–1.52],** ***p* = 0.039**	1.03 [0.84–1.27], *p* = 0.753	1.19 [0.87–1.62], *p* = 0.284	1.23 [0.97–1.55], *p* = 0.084
Thromboembolism						
*IR [95%CI]*	1.0 [0.8–1.2]	1.3 [1.1–1.5]	1.3 [1.1–1.6]	1.6 [1.4–1.9]	1.3 [0.9–1.8]	1.5 [1.2–1.8]
*aHR [95%CI]*	Ref	1.02 [0.78–1.33], *p* = 0.872	1.05 [0.80–1.38], *p* = 0.715	1.24 [0.97–1.59], *p* = 0.089	1.11 [0.74–1.67], *p* = 0.602	1.00 [0.73–1.37], *p* = 1
Major Bleeding						
*IR [95%CI]*	0.8 [0.6–1.0]	1.0 [0.8–1.1]	1.4 [1.2–1.6]	1.3 [1.1–1.5]	1.5 [1.0–2.0]	2.0 [1.7–2.3]
*aHR [95%CI]*	Ref	1.02 [0.75–1.38], *p* = 0.894	1.29 [0.96–1.74], *p* = 0.087	**1.39 [1.04–1.84],** ***p* = 0.024**	**1.65 [1.11–2.46],** ***p* = 0.013**	**1.68 [1.21–2.33],** ***p* = 0.002**

Bold text depicts statistically significant results at *p* < 0·05 level

*aHR* adjusted Hazard Ratio, *CI* Confidence Intervals, *IR* Incidence Rate, *Ref.* Reference

Among the exploratory secondary outcomes, similar results were observed for all-cause mortality, while only the Heart Failure pattern was found associated with an increased hazard of MACE. Risk of major bleeding was found higher in the CV prevention, Mixed Morbidity and High Medicated groups, whereas no statistically significant association was observed between any of the groups and the risk of thromboembolism.

## Discussion

We explored patterns of treatments in a contemporary and global cohort of patients AF. Our main findings were: 1) specific patterns of treatments can be found in patients with AF, reflecting underlying complexity and burden of comorbidities; 2) different treatment patterns were mostly driven by key comorbidities and their interplay, that determined the use of some drugs over others; and 3) these patterns were heterogeneously associated with both use of antithrombotic drugs, and risk of major outcomes, reflecting the differences in the underlying clinical complexity of these patients.

Over recent years, interest on how the contribution of treatment complexity impacts the natural history and prognosis of cardiovascular diseases has grown, including in patients with AF. Nonetheless, most of the research has focused on polypharmacy, using a numerical approach to characterize the burden of pharmacological treatment [4, 5, 16]. Conversely, previous studies have tried to characterize drug use in other clinical contexts, showing heterogeneity in the underlying baseline characteristics of patients, appropriateness of drugs use, and associations with long-term outcomes [7, 17, 18].

With this exploratory analysis, we provide an attempt to move beyond the concept of polypharmacy, emphasizing indeed that different patterns of medication can be found in patients with AF, and that these were heterogeneously associated with the underlying burden of risk factors, OAC use, and the risk of major outcomes.

Of note, we did not consider the use of OAC in patients’ clusterization analysis, to focus on other drugs prescription when exploring patterns of treatments; furthermore, the specific indication for the use of certain cardiovascular drugs (e.g. ACEi/ARBs or beta-blockers) was not available, therefore the patterns were described considering the most relevant characteristics (including comorbidities along with drugs) for each class. Specifically, we found three patterns mostly associated with a cardiovascular fingerprint (the Hypertension, Heart Failure and CV Prevention groups), along with a Low Medicated group, and two other patterns characterized by either a mixed morbidity fingerprint, or a high number of pharmacological treatments, reflecting a higher proportion of cardio-metabolic conditions.

These results expand previous observations on how clinical complexity unfolds in patients with AF [9, 19, 20]. We previously showed how patients with AF could be characterized according to their comorbidities, showing an increasing grade of complexity along with prevalence of both cardiovascular and non-cardiovascular conditions [9], and we also showed how clinical risk factors and determinants of complexity, such as frailty, multimorbidity and polypharmacy, interact in patients with AF and influence their management and prognosis [6, 19].

Herein, we showed how phenotypic patterns of treatment use follow this underlying complexity. This approach may be useful to characterize groups of patients with AF using a physician-oriented perspective. Indeed, the differential use of some treatments observed across different groups may not only reflect the prevalence of diseases, but also the perceived need for specific treatments or for an intensive treatment strategy for a combination of diseases. For example, the overall higher prevalence of both cardiovascular and metabolic conditions (including obesity and diabetes) in the High Medicated pattern can certainly explain the overall higher use of several of the drugs considered in our analysis, and the overall higher prevalence of polypharmacy. On the other side, use of PPI/H2 blockers increased with the complexity of patients, perhaps reflecting a higher likelihood of co-prescription with other drugs. A similar increase was observed for antidepressants, which may reflect a higher psychological morbidity mediated by the increasing complexity of these patients, and reemphasises the importance of considering psychological morbidity in this context [21], especially for those patients with more with more complex clinical profiles.

OACs were largely used across groups (reflecting current increasing trend in use of OAC in patients with AF [22]); notwithstanding this, we found a higher likelihood of receiving OAC in all patterns compared to the Low Medicated one, consistent with the higher thromboembolic risk. Specifically, the highest magnitudes were observed in the Heart Failure, Mixed Morbidity and High Medicated patterns, again suggesting the underlying perceived complexity of these patients. Conversely, we did not observe differences in the use of NOAC vs. VKA across groups at multiple regression analyses, consistent with the steadily increase in adoption of NOACs during the last decade (and the GLORIA-AF Registry Phase III study years) [22].

The higher risk of the primary outcome observed in some of the groups is consistent with the previous observations on the use of OAC. These results are likely explained by the bidirectional effect of patient’s complexity on both treatment patterns and risk of major outcomes, and are further reinforced by the exploratory analyses on the secondary outcomes, and particularly on all-cause mortality.

Conversely, the lack of association with thromboembolic events should be interpreted in the context of a generally high use of OAC, and a broadly low rate of thromboembolism observed; other factors (including a higher dependency of thromboembolic risk to cardiovascular risk factors) may also contribute to explain these results. Together with the increased risk of major bleeding found in the most complex patterns, our findings underscore the differential and heterogenous impact of patient’s complexity on the natural history of patients with AF, also reflecting the ongoing changes in the epidemiology of AF, the higher awareness of thromboembolic risk, and the consequently higher prescription of OAC in clinical practice, along with improvements in the management of the other cardiovascular risk factors. These results, indeed, are consistent with recent epidemiological analyses, that showed a decline in the risk of AF-related thromboembolism over last decades [23, 24]. Conversely, far more conflicting results were observed for mortality and bleeding events, with some reports suggesting a potential increase in the risk of these events [25, 26] and even some evidence for a “decoupling” of cardiovascular and non-cardiovascular mortality, with less positive trends observed for the latter [27].

Our results suggest that the complexity driven by the interplay of cardiovascular and non-cardiovascular conditions can be – at least partly – responsible for the higher risk of mortality and major bleeding observed in our analysis. On the other hand, improvements in prevention of cardiovascular and thromboembolic events may explain the lack of association found for these outcomes in our study.

### Clinical implications

First, characterization of treatment patterns may represent a more granular approach to assess polypharmacy and – more generally – the complexity introduced by medical treatments in patients with AF, compared to the more common approach of counting drugs prescribed or used by the patients. Second, in the context of AF, our results suggest that physicians incorporate the perceived complexity in their clinical decision process on OAC use, even beyond commonly acknowledged thromboembolic risk factors. Finally, our results underscore the need to provide a more comprehensive and holistic care of AF, in view of the evolving and increasingly complex health needs of these patients. This is consistent with recommendations issued by international guidelines for the management of AF [28–30], with the increasing emphasis posed on non-cardiovascular risk factors [31] and with evidence of improved prognosis associated with implementation of an integrated care approach according to the Atrial fibrillation Better Care pathway [32–34], also in patients with multimorbidity [35], polypharmacy [36] and more generally clinically complex patients with AF [6, 37].

In this context, more rational decision-making on the appropriateness of pharmacological treatments, including simplification of treatment schemes and deprescribing approaches, as well as the periodic assessment of treatment indication [38, 39], may have a role in ensure an optimal patient-centred approach to complex patients, with potential benefit on both quality of life and prognosis.

### Strengths and limitations

This analysis is based on a large, multinational and contemporary registry of patients with AF, provides an adequate perspective to explore our research question. Moreover, data on 3-year follow-up were prospectively collected in the GLORIA-AF Registry, allowing for a longitudinal analysis on the risk of long-term outcomes in patients with a recent diagnosis of AF.

Nonetheless, we acknowledge some limitations. First, this is an exploratory analysis aimed at characterizing treatment patterns in patients with AF – as such, this analysis may have been influenced by the data and drugs for which we had available information and that we considered in our analysis (also considering their prevalence of use and data availability). We had more data regarding cardiovascular drugs, while some non-cardiovascular drugs were not available and/or not defined, and therefore our patterns could have been influenced by this imbalance in the granularity of data. Additionally, data on most recent cardiovascular drugs for HF (e.g. sacubitril/valsartan and sodium glucose cotransporter 2 (SGLT2)-inhibitors), treatment adherence and efficacy (e.g. reaching of glycaemic target) were not available, and this poses some limitations to our results. Moreover, treatment patterns could have been influenced by geographical variation and specific time periods. We have restricted our analysis to patients enrolled in the phase III of the GLORIA-AF Registry (2014–2016), so to analyse a narrower period of years; nonetheless, variations in practice and guideline recommendations may have influenced the use of some drugs that we analysed. Moreover, it could not take into account more recent evidence of the importance of an early rhythm control strategy for patients with a recent diagnosis of AF [39] and might not reflect current clinical practice. Finally, although we tried to adjust our multivariable analysis for several covariates, we cannot exclude the further contribution of other unaccounted confounders on the results observed, particularly on the risk of major outcomes. Moreover, our results were not adjusted for multiple comparisons, and as such should be interpreted with caution and as exploratory. For all these reasons, translation of these patterns in clinical practice would require further validation and confirmation in other cohorts. Despite these limitations, we believe that this analysis underlines the importance of considering treatment complexity as another domain of potential intervention in patients with AF, considering patterns of treatment beyond polypharmacy in the decision-making process to tailor management of patients with AF. Future research should be directed in assessing the benefit of the evaluation of prescribed treatment over time to improve outcomes and treatment adherence of patients with AF.

## Conclusions

In patients with AF, specific patterns of drug treatments can be identified, reflecting the underlying clinical complexity and the interaction of some specific comorbidities. These patterns were associated with varying OAC use and with specific long-term clinical outcomes.

## Supplementary Information

Additional file 1: Appendix. List of GLORIA-AF Investigators. Table S1. Metrics for Models from 2 to 6 classes. Table S2. Baseline Characteristics according to latent classes. Figure S1. Antithrombotic prescribed at baseline according to the latent classes allocation.

## Data Availability

Data supporting this study by the data contributors Boehringer Ingelheim, and were made and are available through Vivli, Inc. Access was provided after a proposal was approved by an independent review committee identified for this purpose and after receipt of a signed data sharing agreement.

## Abbreviations

ACEi: Angiotensin converting enzyme inhibitor
ARB: Angiotensin receptor blocker
AF: Atrial fibrillation
BIC: Bayesian Information Criterion
cAIC: Consistent Akaike Information Criterion
CRF: Case report forms
CI: Confidence interval
CV: Cardiovascular
H2: Histamine H2
HR: Hazard Ratio
IQR: Interquartile range
LCA: Latent class analysis
MACE: Major adverse cardiovascular events
NOAC: Non-vitamin K antagonist oral anticoagulants
OAC: Oral anticoagulant
OR: Odds Ratio
PPI: Proton pump inhibitor
SD: Standard deviation
SGLT2: Sodium glucose cotransporter 2
SSRI: Selective serotonin reuptake inhibitor
VKA: Vitamin K antagonist
